# MicroRNAs upregulated during HIV infection target peroxisome biogenesis factors: Implications for virus biology, disease mechanisms and neuropathology

**DOI:** 10.1371/journal.ppat.1006360

**Published:** 2017-06-08

**Authors:** Zaikun Xu, Eugene L. Asahchop, William G. Branton, Benjamin B. Gelman, Christopher Power, Tom C. Hobman

**Affiliations:** 1 Department of Cell Biology, University of Alberta, Edmonton, Alberta, Canada; 2 Department of Medicine, University of Alberta, Edmonton, Alberta, Canada; 3 Department of Pathology, University of Texas Medical Branch, Galveston, Texas, United States of America; 4 Department of Medical Microbiology & Immunology, University of Alberta, Edmonton, Alberta, Canada; 5 Women & Childrens Health Research Institute, University of Alberta, Edmonton, Alberta, Canada; 6 Neuroscience and Mental Health Institute, University of Alberta, Edmonton, Alberta, Canada; 7 Li Ka Shing Institute of Virology, University of Alberta, Edmonton, Alberta, Canada; University of North Carolina at Chapel Hill, UNITED STATES

## Abstract

HIV-associated neurocognitive disorders (HAND) represent a spectrum neurological syndrome that affects up to 25% of patients with HIV/AIDS. Multiple pathogenic mechanisms contribute to the development of HAND symptoms including chronic neuroinflammation and neurodegeneration. Among the factors linked to development of HAND is altered expression of host cell microRNAs (miRNAs) in brain. Here, we examined brain miRNA profiles among HIV/AIDS patients with and without HAND. Our analyses revealed differential expression of 17 miRNAs in brain tissue from HAND patients. A subset of the upregulated miRNAs (miR-500a-5p, miR-34c-3p, miR-93-3p and miR-381-3p), are predicted to target peroxisome biogenesis factors (PEX2, PEX7, PEX11B and PEX13). Expression of these miRNAs in transfected cells significantly decreased levels of peroxisomal proteins and concomitantly decreased peroxisome numbers or affected their morphology. The levels of miR-500a-5p, miR-34c-3p, miR-93-3p and miR-381-3p were not only elevated in the brains of HAND patients, but were also upregulated during HIV infection of primary macrophages. Moreover, concomitant loss of peroxisomal proteins was observed in HIV-infected macrophages as well as in brain tissue from HIV-infected patients. HIV-induced loss of peroxisomes was abrogated by blocking the functions of the upregulated miRNAs. Overall, these findings point to previously unrecognized miRNA expression patterns in the brains of HIV patients. Targeting peroxisomes by up-regulating miRNAs that repress peroxisome biogenesis factors may represent a novel mechanism by which HIV-1 subverts innate immune responses and/or causes neurocognitive dysfunction.

## Author summary

Host cells employ a myriad of antiviral defense systems but most viruses have developed effective countermeasures. Viruses such as HIV that cause lifelong infections are particularly successful in subverting the host antiviral response. While mitochondria have long been known to be critical hubs for antiviral signaling, it has only recently become apparent that peroxisomes are also important for this process. Peroxisomes are small and numerous structures that are best known for their roles in lipid metabolism. New evidence suggests that pathogenic viruses such as West Nile and Dengue viruses block the production of peroxisomes by sequestering and degradation a critical biogenesis factor. In the present study, we report that HIV significantly reduces the number of peroxisomes in infected cells via a completely novel mechanism. Specifically, HIV-infected cells express high levels of microRNAs that inhibit production of proteins required for peroxisome formation. Interestingly, levels of these microRNAs were elevated in the brains of patients with HIV-associated neurocognitive disorders. Thus, as well as affecting antiviral signaling, loss of peroxisomes during HIV infection may contribute to development of neurological disorders. Understanding how pathogenic viruses affect peroxisome biogenesis and cognate antiviral signaling may ultimately lead to novel therapeutic avenues and prevention of long-term sequelae.

## Introduction

Leukocytes infected by human immunodeficiency virus type 1 (HIV-1) traverse the blood-brain barrier within days of primary infection resulting in subsequent infection of macrophage lineage cells (microglia and perivascular macrophages) and astrocytes in the central nervous system (CNS) [1, 2]. As HIV/AIDS progresses, a subset of infected patients develop a neurological syndrome termed HIV-associated neurocognitive disorders (HAND) [3, 4]. HAND affects approximately 25% of HIV-infected patients despite the availability of effective antiretroviral therapy [3, 5–7]. Some of the proposed mechanisms that contribute to HAND include genetic host susceptibility factors, viral properties [8–11] and altered host immune responses [12, 13]. Moreover, neurotoxic effects of some antiretroviral therapies have been implicated in HAND development (reviewed in [14]). The collective actions of neurotoxic viral proteins and chronic neuroinflammation mediated by cytokines and free radicals culminate in synaptic injury and eventual neuronal death, leading to HAND. There are currently no specific therapies for HAND although antiretroviral therapy can alleviate some neurological defects.

Among the factors suggested to contribute to the development of HAND is altered expression of host cell microRNAs (miRNAs). These small noncoding RNAs can regulate both host and viral gene expression [15] and profiling miRNAs in different pathological conditions has yielded important insights into underlying disease mechanisms [16–18]. To this end, it was recently reported that miRNA profiles in the central nervous systems of HIV-infected patients with HAND, differs from nonHAND patients [19, 20]. Similarly, the miRNA signatures in blood from HIV-infected elite controllers differ from those of viremic patients, HAND and nonHAND patients [21–23]. Importantly, altered expression of host miRNAs may not only contribute to the development of HAND but also could potentially be exploited as diagnostic and prognostic biomarkers for HAND [23]. To further investigate the link between host miRNA expression and HAND development as well as HIV-1 biology, brain miRNA profiles were examined in HIV/AIDS patients with and without HAND. We identified 17 miRNAs that had abnormal expression levels in the brains of HAND patients. Bioinformatic analyses revealed that four of the up-regulated miRNAs target key peroxisome biogenesis factors.

Peroxisomes are ubiquitous and essential subcellular organelles responsible for the catabolism of fatty acids (beta oxidation), amino acids, reduction of free radicals such as hydrogen peroxide and the synthesis of plasmalogens. The latter is critical for myelin formation and brain development [24]. Formation of peroxisomes requires multiple peroxin (PEX)-encoding genes and mutations result in devastating diseases that include defects in brain development (reviewed in [25, 26]). In addition to their roles in cellular lipid metabolism and brain development and function, peroxisomes serve as signaling platforms in antiviral defense [27] further underlying their importance in human health. Activation of peroxisomal-MAVS during RNA virus infections leads to the production of type III interferon (IFN) as well as IFN-stimulated genes (ISGs) [27, 28]. Peroxisomes play a role in sensing the HIV-1 genomic RNA [29] and stimulation of peroxisome proliferator-activated receptor alpha by fenofibrate impairs replication of HIV-1 and flaviviruses *in vivo* [30, 31]. Consistent with their roles in antiviral defense, a number of recently published reports revealed that during viral infection, peroxisome biogenesis and/or peroxisome-based signaling is disrupted [32–34]. In these cases, viral proteins directly interact with peroxisomes or biogenesis factors to interfere with peroxisome function or formation.

Here, we show for the first time that peroxisomes are depleted during HIV-1 infection via a unique mechanism. While the PEX mRNA targeting miRNAs were initially discovered in the brains of HIV-infected patients with neurocognitive defects, subsequent analyses revealed that their upregulation is a fundamental aspect of HIV infection. Thus as well as potentially blunting the innate immune response during early stages of infection, HIV-1 induced loss of peroxisomes may play a role in development of neurological disorders in AIDS patients.

## Results

### Brains of patients with HIV-associated neurocognitive disorder (HAND) exhibit distinct microRNA profiles

**Fig 1:**
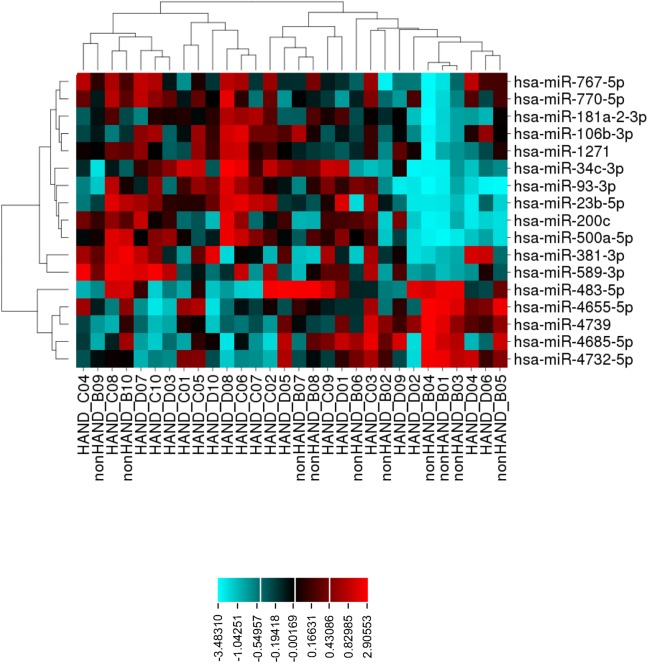
Distinct miRNA profile in brains of HAND patients. 12 up-regulated (red) and 5 down-regulated (blue) miRNAs were identified in brains of HAND (n = 20) compared to nonHAND (n = 10) patients based on Gene Spring RMA normalization method. miRNAs that were down-regulated (bottom right) cluster together while up-regulated miRNAs (top right) form another cluster. Also, those from HAND and non-HAND patient samples form separate clusters.

The development of HAND is dependent on multiple factors including aberrant expression of host-encoded miRNAs. To determine whether there were signature miRNA expression patterns common to HAND patients, we examined a well-defined patient cohort [35–37], focusing on miRNA profiles in brain tissue from HIV/AIDS patients with HAND (n = 20; with encephalitis, n = 10 and without encephalitis, n = 10) to HIV/AIDS patients without HAND or encephalitis (n = 10). To ensure there were sufficient patients in each group and because there were no significant differences in miRNA expression between the two HAND groups, the results from each HAND group were pooled. We found that expression of 17 miRNAs (Fig 1 and Table 1) was consistently dysregulated in the HAND samples. Twelve of the miRNAs were upregulated and five were down-regulated at least 1.5 fold (p < 0.05).

**Table 1:** Potential targets of miRNAs differentially expressed in HAND brain tissue.

miRNA	Expression	Predicted targets						
hsa-miR-4685-5p	Down-regulated	NDOR1	NRAS	PEX14	PRKCA	VDR	IRF1	TAL1
		TK2	PPP3CB	MAVS	FURIN	BCL2		
hsa-miR-4655-5p	Down-regulated	KSR1	AKAP7					
hsa-miR-483-5p	Down-regulated	ALCAM	MAPK3					
hsa-miR-4732-5p	Down-regulated	BCLAF1	CPSF6	CXCL10	GRM5	MKRN3	DHX57	DHX38
hsa-miR-4739	Down-regulated	NFAT5	DDX17	MARK2	NLGN3	KSR2	DHX15	VAPB
		STAT2	CADM3	SYT2	IL6R	CD4	BAK1	OCLN
		DAPK1	CXCR3	NCAN	MYOM1			
hsa-miR-381-3p	Up-regulated	TAOK1	MAVS	CLDN1	ITCH	DDX6	KRAS	VAPA
		**PEX13**	MARK1	NFIA	NFIB	TEK	PCSK2	CXCL3
		XPO7	STAT1	DHX33	DDX52	IL13	CHEK1	CXCR4
		GRM5	DHX40	TTBK2	NLGN1			
hsa-miR-200c-3p	Up-regulated	NFIA	MAP4K5	NLGN4X	DDX3X	DDX3Y	DDX1	DDX53
		DDX55	VAPA	TAOK3	JUN	PCSK2	MAP3K1	CXCL9
		MAP4K3	DCP2	IRF4	TBK1	NUP153	MMP16	XPO1
		IPO7	NOTCH1					
hsa-miR-181a-2-3p	Up-regulated	TAOK1	TAOK3	DDX46	MAPK8	NFAT5		
hsa-miR-1271	Up-regulated	MTSS1	MSN	KRAS	RAB8B	MAP2K1	IRF6	NLGN2
		AK3	MED1	BCL2	TTBK2	TAOK1	LNX2	PRKCE
		CASP2	MRAS					
hsa-miR-770-5p	Up-regulated	MYO6	MAP3K1	NTRK2	DHX15	MARK1	VAPA	
hsa-miR-767-5p	Up-regulated	NFIA	NRAS	SCAI	DDX5	DDX3X	CLDN11	VAPA
		FURIN	OCLN	TAOK2	XPO4	MARK1	KRAS	
hsa-miR-589-3p	Up-regulated	MMP16	CCR9	AAK1	DHX36	MAP3K7	VAPB	
hsa-miR-500a-5p	Up-regulated	IPO9	**PEX2**	MMP8	MMP16	OCLN	CLDN1	TIA1
		CADM2	CCR4					
hsa-miR-106b-2-3p	Up-regulated	IRAK2						
hsa-miR-93-3p	Up-regulated	IRF1	IRF2	FURIN	DAPK1	NFIA	NLGN1	DDX26
		PTEN	DDX5	STAT3	XPO4	CLDN8	**PEX11B**	CD58
		MAVS	NFAT5					
hsa-miR-34c-3p	Up-regulated	NCKAP1	MAP3K2	SCAI	**PEX7**	MARK1	DHX35	DHX9
		EIF4E	MMP24					
hsa-miR-23b-5p	Up-regulated	ADAM10	IL6R	CXCL12	LAMP1	IRF1	IRF2	DHX15
		TLR4	KSR1	DDX6	DDX5	CADM3		

Three algorithms (TargetScan, miRDB and DIANA) were used to predict targets of each miRNA and high-ranking potential targets predicted by at least two out of three algorithms are shown. Notably, peroxisomal genes (PEX2, PEX7, PEX11B and PEX13) that are the predicted targets of 4 up-regulated miRNAs (miR-500a-5p, miR-34c-3p, miR-93-3p, and miR-381-3p) are bolded and underlined.

### Several miRNAs that are deregulated in HAND patients target mRNAs encoding peroxisomal proteins

To understand the potential effects of the differentially expressed miRNAs in pathogenesis of HAND and/or HIV-1 biology, it was important to elucidate their cellular targets. Three bioinformatics algorithms (miRDB, DIANA, and TargetScan) were used to predict potential targets of the 17 differentially expressed miRNAs. We first focused on targets that were predicted by at least two of the three algorithms. In keeping with the notion that a single miRNA can affect expression of dozens of mRNAs, we identified hundreds of potential targets. Some of the highest-ranking candidates are listed in Table 1. Interestingly, four of the up-regulated miRNAs (miR-500a-5p, miR-34c-3p, miR-93-3p, and miR-381-3p) are predicted to target mRNAs encoding the peroxins PEX2, PEX7, PEX11B and PEX13. These proteins play different but critical roles in biogenesis of peroxisomes. Specifically, PEX2 and PEX13 are required for import of peroxisomal matrix proteins; PEX11B facilitates peroxisomal division and proliferation and PEX7 functions as a receptor for the import of peroxisomal matrix proteins with type 2 targeting motifs (reviewed in [38]).

**Fig 2:**
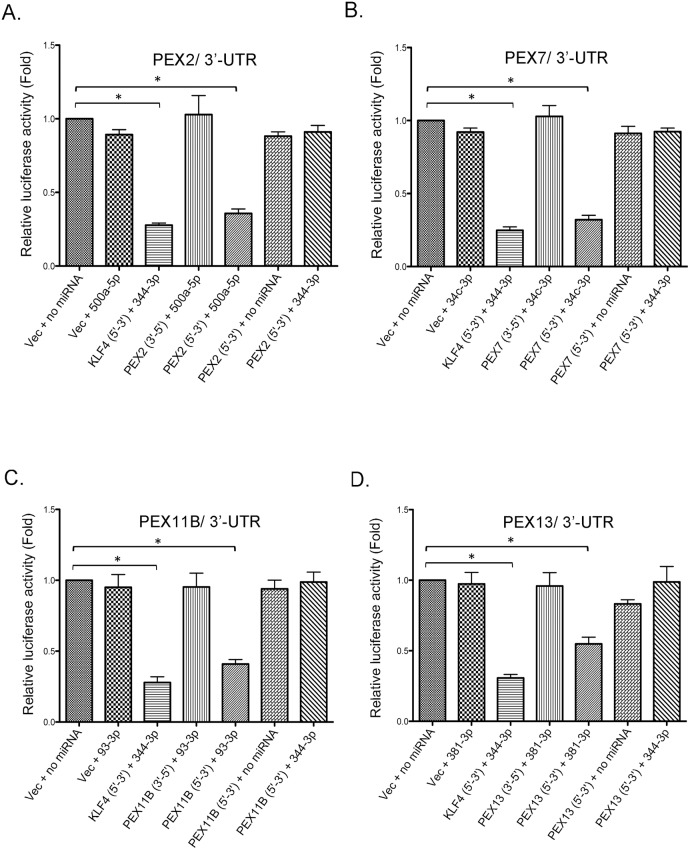
A subset of HAND-associated miRNAs negatively regulate expression of PEX mRNAs. HEK293T cells were co-transfected with luciferase reporter plasmids (pMIR-REPORT-Luciferase) containing 3’-UTRs from PEX2 (A), PEX7 (B), PEX11B (C) and PEX13 (D) in forward (5’-3’) or reverse orientations (3’-5’), a transfection control reporter plasmid (pMIR-REPORT-β-gal) and miRNA mimics for miR-500a-5p, miR-34c-3p, miR-93-3p, miR-381-3p and miR-344-3p. After 48 hours, cell lysates were subjected to luciferase and β-gal assays. N = 3. Bars represent standard error of the mean. Key to plasmids: Vec = pMIR-REPORT-Luciferase; KLF4 = pMIR-REPORT-Luciferase with 3’ UTR of KLF4 downstream from luciferase cassette; PEX2 = pMIR-REPORT-Luciferase with 3’ UTR of PEX2 downstream from luciferase cassette; PEX7 = pMIR-REPORT-Luciferase with 3’ UTR of PEX7 downstream from luciferase cassette; PEX11B = pMIR-REPORT-Luciferase with 3’ UTR of PEX11B downstream from luciferase cassette; PEX13 = pMIR-REPORT-Luciferase with 3’ UTR of PEX13 downstream from luciferase cassette. As a positive control, miR-344-3p is shown to downregulate expression of luciferase under the control of the 3’UTR of KLF4 mRNA.

Peroxisomes have only recently been shown to play roles in antiviral defense [24, 25] but have long been linked to neuroinflammation (reviewed in [39]). As such, we elected to determine if/how the HIV-induced miRNAs affect expression of peroxisomal proteins. In most cases, miRNAs negatively regulate gene expression at the post-transcriptional level through binding to the 3’untranslated regions (UTRs) of mRNAs. Therefore, we first determined whether miR-500a-5p, miR-34c-3p, miR-93-3p, or miR-381-3p affected expression of a reporter gene upstream from the 3’UTRs of PEX2, 7, 11B or 13 mRNAs (Fig 2). The pMIR-REPORT miRNA expression reporter system consists of a firefly luciferase reporter vector (for 3’-UTR cloning) and a β-gal reporter control plasmid (for normalization based on potential differences in cell viability and transfection efficiency). Several controls were included for each experiment. For example, miR-344-3p targets the 3’UTR of KLF4 [40] and therefore, we used this miRNA as the positive control. As a negative control, cassettes encoding the 3’-UTRs for the PEX genes were cloned into the reporter vector in the opposite direction.

Expression of luciferase activity under the control of PEX2, PEX7, PEX11B, or PEX13 UTRs was inhibited by 50–70% in cells transfected with miR-500a-5p, miR-34c-3p, miR-93-3p or miR-381-3p respectively (Fig 2). Conversely, these miRNAs did not affect luciferase activity when the orientations of PEX 3’UTRs were reversed. Together, these data indicate that four of the miRNAs upregulated in the brains of HAND patients efficiently suppress translation of PEX mRNAs.

### Expression of miR-500a-5p, miR-34c-3p, miR-93-3p, and miR-381-3p significantly decrease levels of peroxisomal proteins

**Fig 3:**
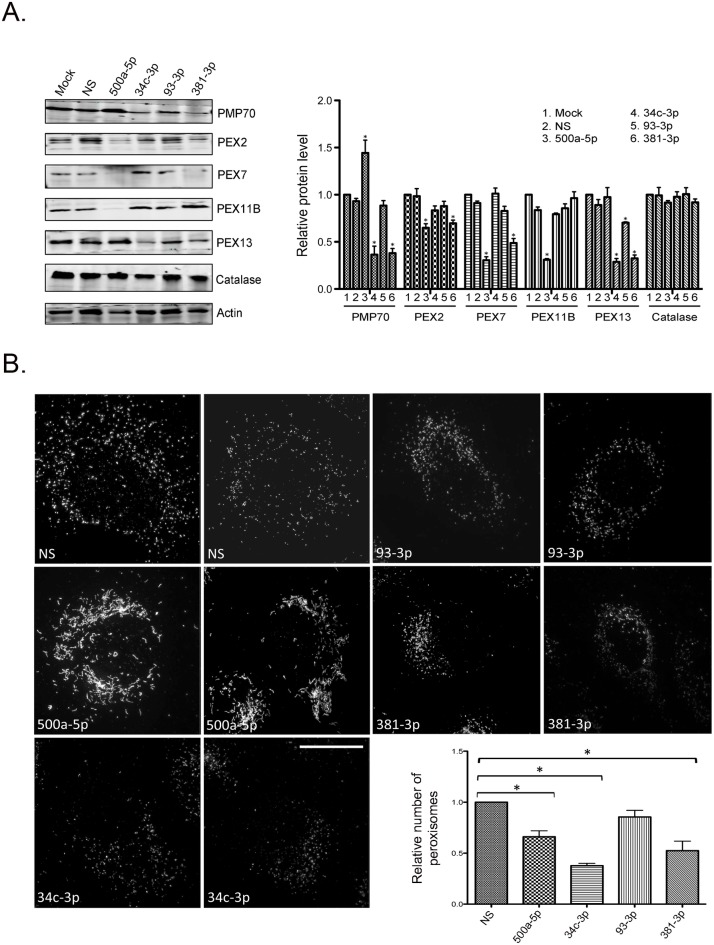
A subset of HAND-associated miRNAs reduces expression of peroxisomal proteins and alters peroxisome abundance and/or morphology. A. A549 cells were transfected with mimics (30 nM) for miR-NS, miR-500a-5p, miR-34c-3p, miR-93-3p or miR-381-3p. Forty-eight hours later, cell lysates were subjected to immunoblot analyses. PMP70 and actin were detected using primary mouse monoclonal antibodies and secondary donkey anti-mouse IgG conjugated to Alexa Fluor 680. PEX2, PEX7, PEX11B, PEX13 and catalase were detected using primary rabbit antibodies and secondary goat anti-rabbit IgG conjugated to Alexa Fluor 680. Relative peroxisomal protein levels (normalized to actin) in mock- and miRNA-transfected cells from three independent experiments are shown. Bars represent standard error of the mean. B. A549 cells were transfected with 30 nM of mimics for miR-NS, miR-500a-5p, miR-34c-3p, miR-93-3p or miR-381-3p for 38 hours after which they were processed for super resolution microscopy. Peroxisomes were identified using a mouse monoclonal antibody to PMP70 and donkey anti-mouse IgG conjugated to Alexa Fluor 488. Nuclei were stained with DAPI. Images were acquired and reconstructed using a DeltaVision OMX structured illumination microscope. Size bar is 10 μM. The relative numbers of peroxisomes in cells transfected with each miRNA were determined using Volocity image analyses software from three independent experiments (minimum of 20 cells). Bars represent standard error of the mean. *, p<0.05.

We next focused on determining whether expression of the PEX mRNA-targeting miRNAs reduced levels of peroxisomal proteins. Immunoblotting was used to quantify the relative levels of peroxisomal proteins in cells transfected with mimics of miR-500a-5p, miR-34c-3p, miR-93-3p, miR-381-3p or a non-silencing miRNA (miR-NS). Data in Fig 3A show that compared to mock and miR-NS-transfected cells, over-expression of miR-500a-5p, miR-34c-3p, miR-93-3p and miR-381-3p resulted in significantly decreased levels of peroxisomal proteins albeit to different extents. Specifically, miR-500a-5p, which targets PEX2 mRNA (Fig 2), reduced levels of PEX2 protein by 35%. Interestingly, PEX7 and PEX11B protein levels were 70% and 69% lower respectively in cells transfected with miR-500a-5p. Similarly, the PEX13-targeting miR-381-3p decreased expression levels of four peroxisomal proteins including PMP70 (a peroxisomal membrane protein), PEX7, PEX13, and PEX2. Unexpectedly, transfection of cells with miR-34c-3p or miR-93-3p mimics did not significantly impact PEX7 or PEX11B protein levels respectively. However, miR-34c-3p expression resulted in loss of both PMP70 and PEX13 proteins. Expression of PEX13 was only slightly decreased by miR-93-3p. Finally, levels of catalase, a peroxisomal matrix protein, were unaffected by expression of the four miRNAs.

There are a number of scenarios in which a single miRNA can affect expression of multiple Pex gene products. One possibility is that miR-500a-5p, miR-381-3p and/or miR-34c-3p inhibit translation of multiple mRNAs that encode PEX proteins. Indeed, miRNAs that target components of a cellular pathway can be synthesized as a common transcript that contains multiple primary miRNAs [41]. However, a search of the miRBase database indicated that genes encoding miR-500a-5p, miR-34c-3p, miR-93-3p, and miR-381-3p are located on different chromosomes. Moreover, the initial miRNA target search using miRDB, DIANA, and TargetScan did not indicate that multiple PEX mRNAs are targeted by miR-500a-5p, miR-34c-3p, miR-93-3p, or miR-381-3p. Nevertheless, we employed the luciferase-based reporter assay described above to experimentally determine if any of these miRNAs could target more than one PEX gene. Data in S1 Fig confirmed that the miRNAs only regulated expression of luciferase under the control of 3’UTRs from their predicted PEX mRNA targets. Specifically, miR-500a-5p, miR-34c-3p, miR-93-3p and miR-381-3p downregulated expression of luciferase under the control of the 3’UTRs from PEX2, PEX7, PEX11B and PEX13 mRNAs respectively.

We also used siRNAs to determine if decreasing expression of PEX2, PEX7, PEX11B or PEX13 proteins affected steady state levels of one another. Unlike miRNAs, which are inherently degenerate with respect to mRNA targets, siRNAs are perfectly complementary to their mRNA targets. siRNAs against PEX2, PEX7, PEX11B or PEX13 were transfected into HEK293T cells and levels of proteins were determined by immunoblotting (S2 Fig). These experiments showed that targeted knockdown of a single PEX protein can indeed result in concomitant loss of other PEX proteins. For example, siRNAs against PEX7 not only reduced the level of PEX7 protein, but PEX11B was also markedly lower. Similarly, a PEX13-specific siRNA reduced the levels of PEX13 and PEX7 proteins. Finally, we showed that downregulation of the multifunctional peroxisome biogenesis factor PEX19 using siRNA, effectively reduced levels of PEX19, PEX7, PEX11B and PEX13 proteins. Unfortunately, we were unable to achieve significant reduction of PEX2 protein with siRNAs, despite using at least three different siRNAs.

### Expression of miR-500a-5p, miR-34c-3p and miR-381-3p dramatically affects peroxisomes

Next we examined how overexpression of miR-500a-5p, miR-34c-3p, miR-93-3p, and miR-381-3p affected peroxisomes. Super-resolution microscopy was used to analyze the morphology, distribution and numbers of peroxisomes in miRNA-transfected cells. Peroxisomes were identified using an antibody to PMP70, a peroxisomal membrane protein involved in membrane assembly [42]. Cells transfected with a non-silencing miRNA (miR-NS) contained hundreds of PMP70-positive puncta throughout the cytoplasm (Fig 3B). While the number of peroxisomes was significantly reduced by expression of miR-500a-5p (which targets PEX2), most striking was the change in morphology and PMP70 staining of the peroxisomes. Specifically, miR-500a-5p over-expression resulted in enlargement and elongation of peroxisomes. Decreasing the intracellular level of PEX2, an E3 ubiquitin ligase that targets PMP70 [43] could certainly explain the higher levels of PMP70 protein (Fig 3A) and increasing staining intensity of anti-PMP70 in miR-500a-5p over-expressing cells (Fig 3B).

It is important to point out that PEX11B is required for peroxisome fission (reviewed in [38]) and as such, the fact that miR-500a-5p expressing cells have lower levels of this protein could result in decreased fission of peroxisomes and concomitant lengthening and enlargement of these organelles. Unexpectedly, the effect of miR-93-3p (which targets PEX11B) on peroxisomes was minimal. Despite evidence showing that the 3’UTR of PEX11B is targeted by this miRNA (Fig 2), PEX11B protein levels were not significantly affected by over-expression of a miR-93-3p mimic (Fig 3A). One possibility is that PEX11B protein is very stable and the cellular pool was not depleted within the time frame of our experiments. Finally, it can be seen that expression of miR-34c-3p and miR-381-3p reduce peroxisome numbers by 65% and 45% respectively (Fig 3B). Notably, this is consistent with the immunoblot data in Fig 3A showing that levels of PMP70 protein were reduced by expression of miR-34c-3p and miR-381-3p.

### HIV-1 infection downregulates peroxisomal proteins and decreases peroxisome numbers

**Fig 4:**
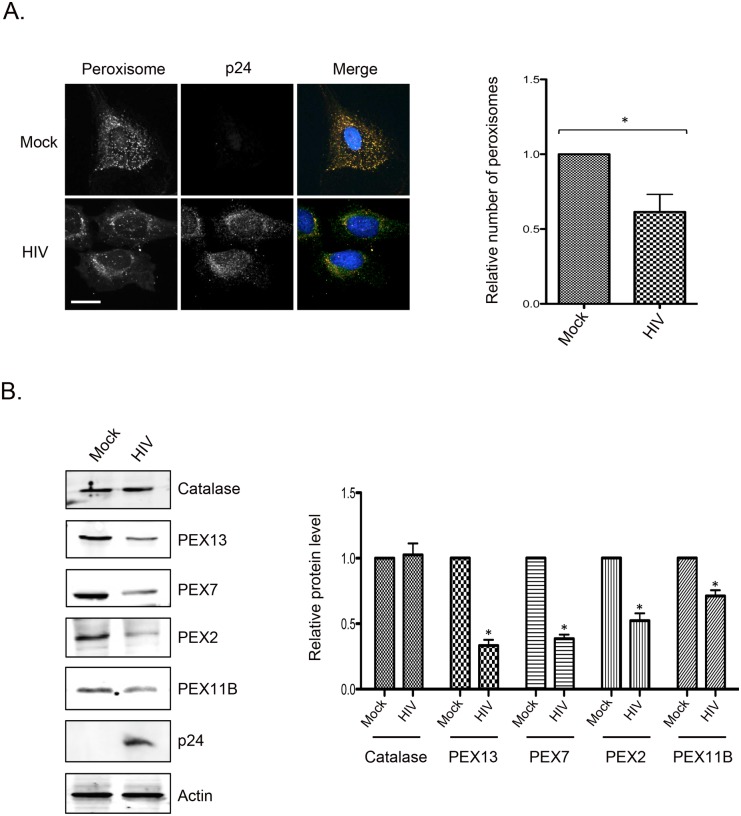
HIV-1 infection causes loss of peroxisomal proteins and reduces the abundance of peroxisomes. A. Hela CD4+ cells (clone 1022) were infected with HIV-1 (pYu2, MOI = 10.0) for 72 hours and then processed for indirect immunofluorescence and confocal microscopy. Peroxisomes were detected with a rabbit polyclonal antibody to peroxisomal targeting signal SKL and donkey anti-rabbit IgG conjugated to Alexa Fluor 546. HIV-infected cells were detected with a mouse monoclonal antibody to HIV-1 p24 protein and donkey anti-mouse IgG conjugated to Alexa Fluor 488. Nuclei were stained using DAPI. Images were obtained using spinning disc confocal microscopy. The numbers of peroxisomes (SKL-positive structures) in mock- and HIV-infected cells were determined using Volocity image analyses software. Averages were calculated from three independent experiments in which a minimum of 10 cells for each sample were analyzed. The average number in mock-treated cells was normalized to 1.0. Bars represent standard error of the mean. * p<0.05. B. Primary monocyte-derived macrophages (MDM) were infected with HIV (pYu2, MOI = 2.0) for 5 days and then were subjected to immunoblot analyses with antibodies to Catalase, PEX2, PEX7, PEX11B, PEX13, HIV p24 and actin. The relative levels of peroxisomal proteins (compared to actin) from 3 independent experiments (3 donors) were averaged and plotted. Error bars represent standard error of the mean. * p<0.05.

To determine if peroxisomes were affected by HIV-1 infection, immunofluorescence and immunoblot assays were conducted on infected Hela CD4+ cells and monocyte-derived macrophages respectively. Data in Fig 4A show that similar to what was observed in miRNA-transfected cells (Fig 3B), HIV infection results in significant loss of peroxisomes in Hela CD4+ cells. These cells were used for the microscopy assays because their flat morphology is more conducive for peroxisome quantitation. Peroxisomes were identified using an antibody to the tripeptide Ser-Lys-Leu (SKL), a targeting motif found at the carboxyl termini of many peroxisomal matrix proteins [44] (Fig 4A). Quantification of SKL-positive structures showed that on average HIV-infected cells contained 40% less peroxisomes than mock-treated cells (Fig 4A). Immunoblotting revealed that infection of primary macrophages, a physiologically relevant cell type in HIV patients, resulted in dramatic loss of PEX2, PEX7, PEX13, and to a lesser extent, PEX11B (Fig 4B). However, levels of catalase, a peroxisomal matrix protein were not affected by HIV infection. This indicates that the effects of HIV-1 protein expression on peroxisome-associated proteins are highly specific. Similar results were observed in infected Hela CD4+ cells (S3 Fig).

**Fig 5:**
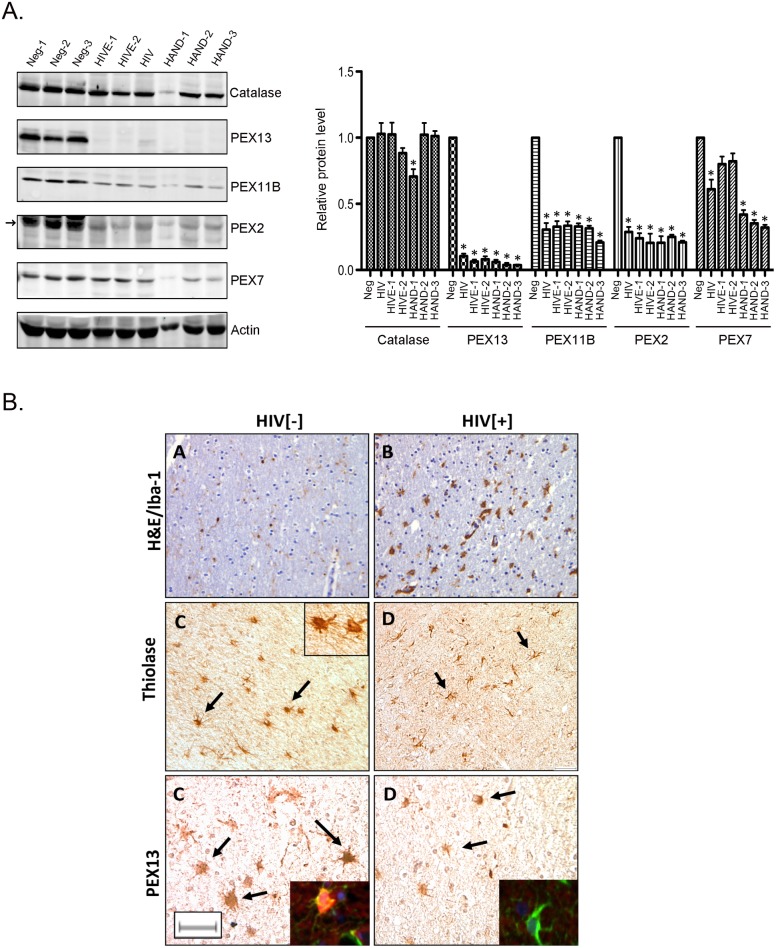
HIV-1 induces loss of peroxisomal proteins in brain tissue. A. Lysates from brain tissue from HIV negative (Neg-1-3), HIV positive (HIV), HIV positive with encephalitis (HIVE-1-2) and HAND patients (HAND-1-3) were subjected to immunoblotting with antibodies to catalase, PEX2, PEX7, PEX11B, PEX13 and actin. The relative levels of peroxisomal proteins (compared to actin) were averaged and plotted. N = 3 (triplicate from same sample). Error bars represent standard deviation of the mean. * p<0.05. B. Immunodetection peroxisome proteins in frontal lobe material from uninfected (HIV[-]) and HIV-infected (HIV[+]) patients. Peroxisomes were labeled with rabbit antibodies PEX13 or thiolase and microglia were detected using rabbit anti-Iba-1. Most of the cells that stain intensely for thiolase and PEX 13 immunopositive cells are astrocytes (arrows) but some could be oligodendrocytes or microglia. Confocal microscopy shows labeled astrocytes (green) and PEX immunoreactivity (scarlet) and DAPI-labeled nuclei. Slides from 4–5 patients per group were reviewed; all HIV+ patients were AIDS-defined and not receiving therapy at the time of death. (Size bar = 20μm).

Next, we used immunoblotting to analyze peroxisomal protein levels and immunohistochemistry to assess peroxisome morphology in frontal lobe brain tissue from HIV/AIDS and uninfected patients. Data in Fig 5A show that PEX13 protein was virtually absent in HIV patients with or without encephalitis or HAND. Levels of PEX7 protein were also significantly (40%) lower in the sample from an HIV patient without encephalitis or HAND, however in three HAND samples, steady state levels of PEX7 protein were lower than those seen in HIV patients without HAND as well as non-infected patients. Finally, levels of PEX2 and PEX11B proteins were reduced (~70–80%) in brain tissue from all of the HIV patients assayed.

As a secondary assay, brain tissue samples from uninfected and HIV/AIDS patients were examined by immunocytochemistry. Immunolabeling of frontal lobe sections showed that the intensity of PEX13 and thiolase immunostaining which was concentrated in astrocytes (arrows), was consistently lower in HIV/AIDS tissue compared to that from uninfected patients (Fig 5B). Although the data are from a small sample size, they suggest that HIV infection contributes to loss of peroxisomal material in brain tissue.

### The four PEX mRNA targeting miRNAs are upregulated during HIV infection of macrophages

**Fig 6:**
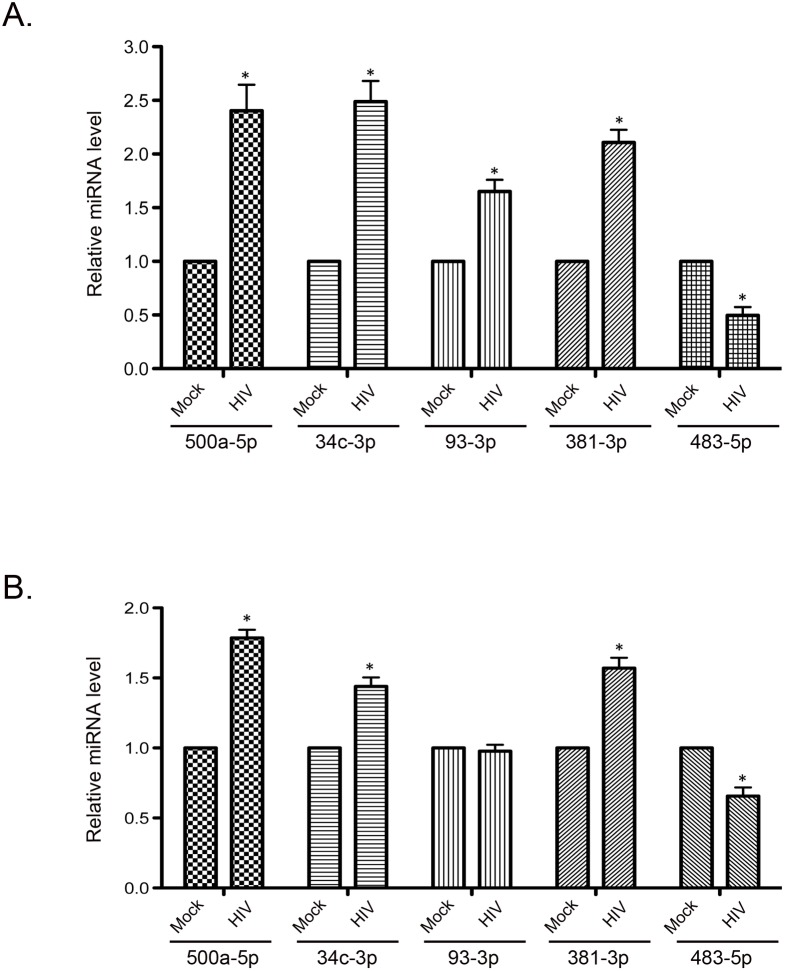
HIV-1 infection upregulates expression level of multiple miRNAs that target PEX mRNAs. A. Primary monocyte-derived macrophages (MDM) from 3 donors were infected with HIV (pYu2, MOI = 2.0) for 5 days and relative levels of miRNAs were determined by RT-PCR from total RNA extracted from the samples. The average relative levels of miRNAs (normalized to snRNU6) from 3 independent experiments were determined. Error bars represent standard error of the mean. B. Hela CD4+ cells were infected with HIV-1 (pYu2, MOI = 10.0) for 48 hours and relative levels of miRNAs were determined as described in panel A. N = 3. Error bars represent standard error of the mean. * p<0.05.

Our data are consistent with a scenario in which the loss of peroxisomes during HIV-1 infection is caused by increased expression of miRNAs that target mRNAs encoding peroxisome biogenesis factors. To address this hypothesis, we first determined if miR-500a-5p, miR-34c-3p, miR-93-3p and/or miR-381-3p were upregulated in HIV-infected macrophages. Human primary macrophages were infected with HIV-1 (MOI = 2) and after 5 days, relative levels of miRNAs were determined by RT-qPCR. Data in Fig 6A show that levels of miR-500a-5p and miR34c-3p were increased almost 2.5 fold whereas miR-93-3p and miR-381p were increased between 1.6 and 2.2 fold. In contrast, levels of miR-483-5p (which does not target PEX mRNAs and was identified as a miRNA whose expression was decreased in brain tissue of HAND patients, Table 1) were slightly decreased in HIV-infected macrophages.

**Fig 7:**
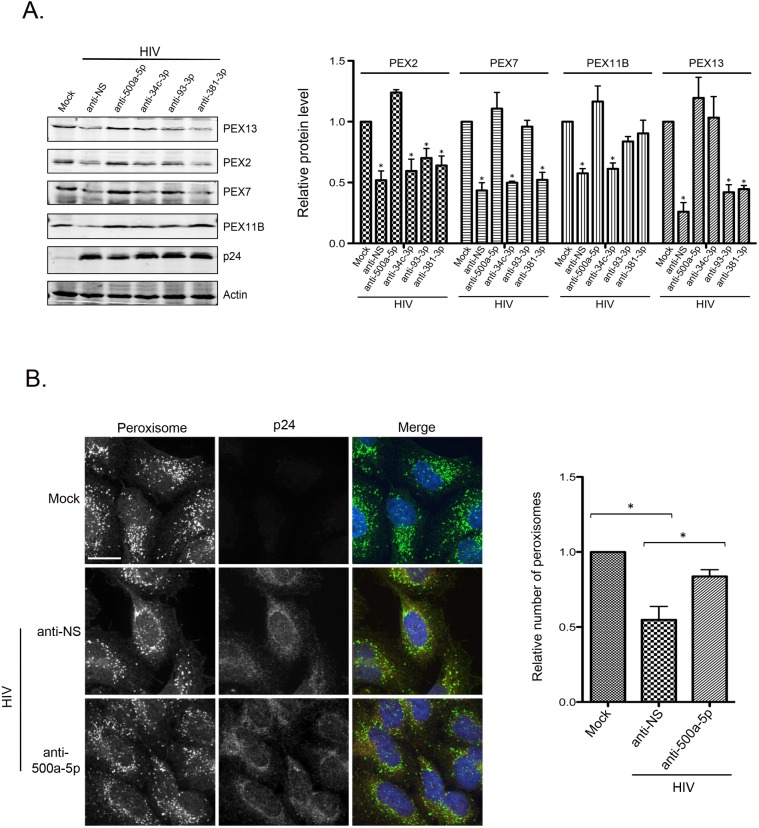
HIV-induced loss of peroxisomal proteins and peroxisomes is abrogated by blocking the function of miR-500a-5p. A. HEK293T cells were transfected with a plasmid encoding HIV-1 provirus (pYU2) for 12 hours after which cells were transfected with anti-miRs that are complementary to the HAND-associated PEX-specific miRNAs. Cell lysates were collected 36 hours later and then subjected to immunoblot analyses with antibodies to PEX2, PEX7, PEX11B, PEX13, HIV-1 p24 and actin. The relative levels of peroxisomal proteins (normalized to actin) from 3 independent experiments were determined. Error bars represent standard error of the mean. * p<0.05. B. Hela CD4+ cells (clone 1022) were infected with HIV-1 (MOI = 10) for 16 hours and then transfected with anti-miR-500a-5p. Forty-eight hours later, cells were processed for indirect immunofluorescence and confocal microscopy. Peroxisomes were detected with a rabbit polyclonal antibody to the peroxisomal targeting signal SKL and donkey anti-rabbit IgG conjugated to Alexa Fluor 488. HIV-infected cells was detected with a mouse monoclonal antibody to HIV-1 p24 and donkey anti-mouse IgG conjugated to Alexa Fluor 546. Nuclei were stained using DAPI. Images were obtained using spinning disc confocal microscopy. Size bar is 10 μM. The relative numbers of peroxisomes (SKL-positive structures) in mock and HIV-infected cells transfected with or without anti-miR-500a-5p were determined using Volocity image analyses software. The average numbers of peroxisomes/cell were calculated from three independent experiments in which a minimum of 10 cells for each sample were analyzed. * p<0.05.

To further investigate the mechanism underlying HIV-associated loss of peroxisomes, we used anti-miRs to block the functions of PEX mRNA-targeting miRNAs during HIV infection. As transfection of primary macrophages can be technically challenging [45], we elected to employ CD4+ Hela cells for these experiments. Data in Fig 6B show that with the exception of miR-93-3p, expression of PEX-targeting miRNAs was significantly elevated in HIV-infected CD4+ Hela cells. Anti-miR-500a-5p had the most dramatic effect in that it completely prevented HIV-induced loss of PEX2, PEX7, PEX11B and PEX13 (Fig 7A). Other miRNA inhibitors had intermediate effects. For example, anti-miR-34c-3p increased levels of PEX13; anti-miR-93-3p increased levels of PEX7 and PEX11B; and anti-miR-381-3p increased levels of PEX11B.

Since miR-500a-5p had the greatest effect on peroxisomal protein expression, we questioned whether blocking the activity of this miRNA could prevent HIV-induced loss of peroxisomes. Results in Fig 7B show that anti-miR-500a-5p abrogated the effect of HIV-1 infection on peroxisomes. Specifically, the average number of peroxisomes in HIV-infected cells containing the inhibitor of miR-500a-5p was not statistically different from that of mock-treated cells.

### Some HIV-induced miRNAs that target PEX mRNAs enhance expression of innate immune genes

**Fig 8:**
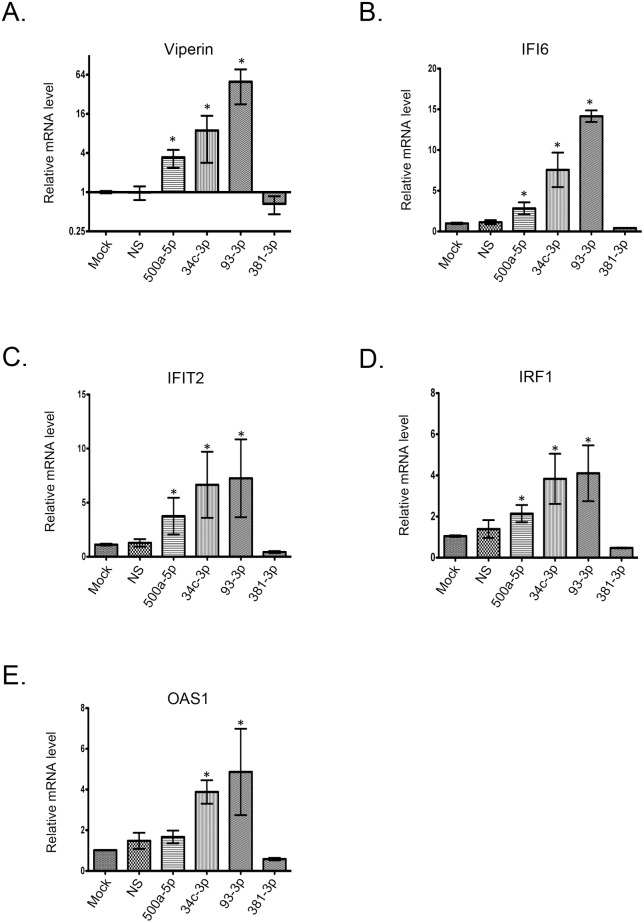
Transfection of miRNA mimics that target PEX mRNAs leads to increased levels of innate immune mRNAs. A549 cells were transfected with miRNA mimics (30 nM) for miR-NS, miR-500a-5p, miR-34c-3p, miR-93-3p or miR-381-3p. Forty-eight hours later, total RNA was extracted from the cells for use in RT-PCR. Relative levels of innate immune mRNAs (Viperin (A), IFI6 (B), IFIT2 (C), IRF1 (D), and OAS1 (E)) from 3 independent experiments were determined by RT-PCR from total RNA extracted from the samples. Error bars represent standard error of the mean.

Because peroxisomes are now recognized to have important roles in antiviral signaling [27, 28], we questioned whether expression of miRNA mimics that target PEX mRNAs would affect innate immune genes. A549 cells were chosen for these experiments because they are human in origin and have been used extensively to study innate immune signaling. Interestingly, three of the miRNA mimics (miR-500a-5p, miR-34c-3p and miR-93-3p) significantly increased mRNA levels for five innate immune genes (Fig 8). MiR-93-3p had the most dramatic affect on expression of antiviral genes. Specifically, in cells transfected with miR-93-3p mimic, expression of IFI6 and viperin mRNAs were increased 14-fold and 50-fold respectively. MiR-500a-5p appeared to modestly increase expression of innate immune genes (2–4 fold) whereas miR-381-3p did not significantly affect expression of viperin, IFI6, IFIT2, IRF1 or OAS1 (Fig 8).

## Discussion

A growing body of research has linked changes in miRNA expression to pathogenesis of neurodegenerative diseases including Alzheimer’s disease, Parkinson’s disease, Huntington’s disease and amyotrophic lateral sclerosis (reviewed in [46]). The original goal of the present study was to identify miRNAs that are differentially expressed in the brains of HIV-infected patients with HAND. Of the 17 miRNAs whose expression levels were commonly deregulated in HAND patients, four (miR-500a-5p, miR-34c-3p, miR-93-3p, and miR-381-3p) were shown to regulate expression of the peroxisome biogenesis factors PEX2, PEX7, PEX11B and PEX13. Subsequent analyses revealed that elevated expression of these miRNAs was not specific to HIV-HAND but rather, was a common feature of HIV infection. This is the first report to our knowledge demonstrating that viral infection leads to increased expression of mRNAs that downregulate peroxisomes, possibly as a mechanism to alter early antiviral signaling that emanates from these organelles.

The present study also connects two divergent areas, HIV-1 biology and peroxisome dysfunction in the brain, providing previously unrecognized insights into the pathogenesis of a common neurological syndrome, HAND. Although larger samples sizes and further analyses are required to confirm our findings, in general, the relative loss of PEX proteins in brain tissue appears to be greater in HAND compared to non-HAND HIV. This scenario is consistent with our initial observation that miR-500a-5p, miR-34c-3p, miR-93-3p, and miR-381-3p were expressed at higher levels in brain tissue from HIV-HAND vs HIV-non-HAND patients. Moreover, a large number of studies indicate that peroxisomes are critical for brain function (reviewed in [24]); thus stressing the need for further investigation into peroxisomes and HIV pathogenesis.

Peroxisome-based diseases in humans can be classified into two large groups; peroxisome biogenesis disorders and single peroxisome enzyme deficiencies. The first group includes Zellwegger syndrome spectrum disorders and rhizomelic chondrodysplasia punctata type 1. In cells from patients with peroxisome biogenesis disorders, peroxisomes are absent due to mutations in one or more genes that encode critical biogenesis factors including PEX2, PEX7 and PEX13 (reviewed in [26]). Not surprisingly, these disorders often result in death at an early age and patients suffer from a wide variety of neurological abnormalities including leukodystrophy (inflammatory degeneration of white matter), similar to that observed in advanced HAND, termed HIV-associated dementia [47]. Mutations in genes that encode peroxisomal enzymes also result in severe neurological deficits, again defined by inflammatory degeneration of white matter [48]. These studies underscore the fact that even partially diminished function of peroxisomes can lead to severe neurological disease.

The function of peroxisomes in antiviral signaling is a relatively new discovery [27, 28]. A pool of the mitochondrial antiviral signaling protein (MAVS), an adaptor protein for retinoic acid-inducible gene 1 protein (RIG-I), localizes to peroxisomes. Activation of MAVS-dependent signaling from peroxisomes by different RNA viruses leads to activation of type III interferon, a process that is thought to complement the type I interferon response induced from mitochondria, which occurs later. MAVS signaling from both peroxisomes and mitochondria is required for maximal anti-viral activity. The observation that viruses have evolved strategies to interfere with peroxisome-dependent anti-viral signaling illustrates the importance of this organelle in defending against pathogens. For example, the hepatitis C virus NS3-4A protease has been shown to cleave both peroxisomal and mitochondrial MAVS to suppress RIG-I signaling of immune defenses [33, 49, 50]. Another mechanism used by flaviviruses such as West Nile virus and Dengue virus, involves targeting of PEX19, a critical peroxisome biogenesis factor, for degradation [32]. Flavivirus-infected cells contain significantly lower numbers of peroxisomes, an effect that is mediated in large part through binding of capsid protein to PEX19. As a result of the reduced peroxisome pool, type III interferon expression is dramatically reduced. Finally, the observation that human cytomegalovirus HCMV protein vMIA, which inhibits signaling downstream from mitochondrial MAVS, also localizes to peroxisomes [34], may indicate that peroxisomes play a role in defense against DNA viruses too. While vMIA interacts with peroxisomal MAVS and induces peroxisome fragmentation, disruption of peroxisomal morphology is not essential for this viral protein to inhibit antiviral signaling. Association of vMIA with peroxisomes may require interaction with PEX19. Although it was not further investigated, it is intriguing to note that MAVS is also a predicted target of miR-93-3p (Table 1).

Here we show that infection by HIV-1, a lentivirus, negatively impacts peroxisomes by a novel mechanism. The fact that blocking miRNA function with anti-miRs abrogates HIV-induced loss of peroxisomes suggests that upregulation of miRNAs is the main mechanism by which the virus targets these critical organelles that function in antiviral defense and neuroprotection. Of note, three of the four PEX proteins (PEX2, PEX7 and PEX13 targeted by HIV-induced miRNAs are associated with peroxisome biogenesis disorders [26]. Interfering with peroxisome biogenesis/function by altering miRNA expression appears to be a very efficient mechanism because some of the HIV-induced miRNAs repress expression of multiple PEX proteins. For instance, miR-500a-5p, which targets PEX2, also reduced expression of PEX7 and PEX11B proteins. Similarly, the PEX13-targeting miR-381-3p decreased expression of PMP70, PEX7, PEX13, and PEX2. This was not due to the miRNAs targeting multiple PEX mRNAs but rather, it seems that expression and/or stability of given PEX proteins is often dependent on other PEX proteins. For example, cells transfected with siRNAs against PEX7 not only reduced PEX7 protein but PEX11B was also markedly lower. Similarly, a PEX13-specific siRNA reduced the levels of PEX13 and PEX7 proteins. Consistent with these data, it has been reported that siRNAs against PEX7 also reduce levels of PMP70 protein [51] and knockout of the PEX2 gene in mice negatively impacts PEX14, PEX3, PEX16 and catalase [52]. Finally, PEX11B and PEX13 knockout mice express lower levels of PEX14 protein [53, 54].

Inhibition of peroxisome biogenesis and/or function during virus infection is only a recently discovered phenomenon [32–34]. However, given the roles of these organelles in antiviral defense and nervous system function, understanding how viruses manipulate peroxisomes will undoubtedly reveal pathological mechanisms that underlie multiple viral diseases. In the case of HIV-1, elevated expression of PEX gene-targeting miRNAs was initially detected among patients with HAND. However, the fact that these miRNAs are also upregulated during HIV-1 infection of macrophages and that loss of peroxisomal proteins was observed in the brains of HIV patients without HAND indicate that virus-induced loss of peroxisomes is a fundamental aspect of HIV biology.

Of interest is whether the degree of PEX-specific miRNA expression correlates with disease severity. The higher levels of PEX-specific miRNA expression in HAND patients compared to HIV patients without HAND is consistent with this scenario. Moreover, in a small sample size, we observed that loss of certain PEX proteins (PEX) was more pronounced in brain tissue from HAND patients compared to HIV patients without HAND. However, further investigation is required to determine if this is a ubiquitous phenotype in HAND patients. It is important to point out that only a small subset of permissive brain cells exhibit detectable viral genome or protein expression. Thus, it is plausible that the miRNA changes as well as altered Pex gene expression in HAND brains might be due in part to effects on bystander cells such as astrocytes, which rarely exhibit *in vivo* productive infection but are the most populous cell type in the brain. Nonetheless, miRNAs are being explored as diagnostic and prognostic biomarkers for various neurological conditions including Alzheimers’s and Parkinson’s diseases as well as HAND (reviewed in [55]).

Presently, there is very little known about aberrant miRNA expression and peroxisomal biogenesis disorders. However, a large number of recent studies have focused on the relationship between miRNA expression and peroxisome proliferator-activated receptors [56–59] and some of the findings have implications for neurological disease. Future studies that further clarify how viruses manipulate miRNAs are likely to reveal novel roles for miRNAs in peroxisome-dependent anti-viral defense, lipid metabolism and neurodegenerative disorders.

## Materials and methods

### Ethics statement

Brain tissue from HAND and non-HAND patients was collected at autopsy with informed consent at different geographical locations (Texas, New York, San Diego and Los Angeles) by the National NeuroAIDS Tissue Consortium [36, 37]. The use of autopsied brain tissues (Protocol number 2291) is approved by the University of Alberta Human Research Ethics Board (Biomedical) and written informed consents from all participants were signed before or at the collection times. The protocols for obtaining post-mortem brain samples comply with all federal and institutional guidelines with special respect for the confidentiality of the donor's identity.

### MicroRNA extraction from human brain tissue

Neocortical brain tissue samples from midfrontal gyrus were excised from fresh-frozen brain slices and shipped in dry ice to the Laboratory for Neurological Infection and Immunity Brain Bank at University of Alberta. Samples were stored at -80°C until total RNA extraction including microRNAs (miRNAs) was performed as follows. Briefly, ~100 mg/sample of autopsy-derived brain tissue was aseptically collected using sterile instruments into a 2 ml Lysing Matrix tube (MP Biomedicals, Santa Ana, CA, USA). Tissue samples were homogenized in a FastPrep-24 tissue homogenizer (MP Biomedicals, Santa Ana, CA, USA) after adding 1 ml of Trizol reagent (Invitrogen Carlsbad, CA, USA). Chloroform (200 μl) was added to each homogenate which was then centrifuged 12,000 x g for 15 minutes at 4°C. The aqueous phase was collected and extraction followed as indicated in the manufacturer’s manual (Qiagen, Catalog no. 217004).

### MicroRNA microarray and statistics analyses

Affymetrix miRNA 3.0 GeneChips were used for miRNA analyses. This microarray chip provides comprehensive coverage for mature human miRNAs (1733 probes) and pre-miRNAs (1658 probes). The Affymetrix FlashTag Biotin highly sensitive and reproducible (HSR) RNA Labelling kit was used to label RNA samples for analysis. Equal concentrations of total RNA including microRNAs (800–1000 ng) were poly-A tailed as specified by the manufacturer (Affymetrix) followed by biotin-HSR ligation. Next, samples were treated with T4 DNA ligase before they were hybridized to Affymetrix miRNA 3.0 GeneChip arrays at 48°C for 16 hours. Arrays were then stained and washed on an Affymetrix GeneChip Fluidics 450 following manufacturer’s protocol and then scanned with an Affymetrix GeneChip Scanner 3000 7G System.

Genespring (version 12.6) software (Agilent Technologies) was used to normalize the data and identify differentially expressed miRNAs. The normalization in this software is based on the Robust Multi-array Average (RMA) algorithm, in which data are background-corrected, log2 transformed and quartile normalized. To identify differentially expressed miRNAs, the median of each probe set in the HAND or nonHAND patients was calculated and the non-parametric test Mann-Whitney unpaired test was applied. To select for differentially expressed miRNAs in this analysis, a cut-off fold change (≥ 1.5) in relative miRNA abundance and a *p* value of <0.05 was considered statistically significant.

### Prediction of microRNA targets

Three different bioinformatics algorithms (miRDB, http://mirdb.org/miRDB/index.html; Diana-microT-CDS;http://diana.imis.athena-innovation.gr/DianaTools/index.php?r=microT_CDS/index; and TargetScanHuman v6.2, http://www.targetscan.org/) were used to predict the potential targets of differentially expressed miRNAs. Only mRNA targets that were predicted by at least two of the three algorithms were investigated further.

### Reagents

Complete EDTA-free protease inhibitor cocktail (Roche Diagnostics (Laval, Quebec, Canada); ProLong Gold anti-fade reagent with 4,6-diamidino-2-phenylindole (DAPI), SlowFade Gold reagent mounting media, cell culture media DMEM, RPMI 1640, and fetal bovine serum (FBS) from Invitrogen (Carlsbad, CA) were purchased from the indicated suppliers.

Lipofectamine 2000 and Lipofectamine RNAiMAX were purchased from Invitrogen (Carlsbad, CA); Per-Fectin transfection reagent was from Genlantis (San Diego, CA).

miRIDIAN microRNA mimics including human hsa-miR-500a-5p, hsa-miR-34c-3p, hsa-miR-93-3p, hsa-miR-381-3p; miRIDIAN microRNA Mimic Negative Control #1 and miRIDIAN microRNA mimic mouse mmu-miR-344-3p; miRIDIAN microRNA inhibitors including human hsa-miR-500a-5p-Hairpin Inhibitor, hsa-miR-34c-3p-Hairpin Inhibitor, hsa-miR-93-3p-Hairpin Inhibitor and hsa-miR-381-3p-Hairpin Inhibitor were purchased from GE Healthcare Dharmacon Inc. (Lafayette, CO).

MGC human PEX2 (Clone ID: 3347824), PEX7 (Clone ID: 5176358), PEX11B (Clone ID: 3866690), and PEX13 (Clone ID: 6285875) sequence-verified full-length cDNA clones were purchased from GE Healthcare Dharmacon Inc. (Lafayette, CO).

Reagents for purification and quantitation of miRNAs including MiRNeasy Mini kit, miScript PCR Starter kit, miScript II RT kit, and miScript SYBR Green PCR kit were purchased from Qiagen (Toronto, ON).

### Antibodies

Mouse monoclonal antibodies against the peroxisomal membrane protein PMP70 (Sigma, St. Louis, MO), HIV-1 p24 (Abcam, Cambridge, MA), and beta-actin (Abcam, Cambridge, MA) were purchased from indicated suppliers. Rabbit polyclonal antibodies to PEX7, PEX11B, PEX13, PEX19 and catalase were from Abcam (Cambridge, MA); Rabbit polyclonal antibody to PEX2 (PXMP3) was purchased from Pierce (Rockford, IL); Rabbit polyclonal antibody to thiolase (ACAA1) was from MyBioSource (San Diego, CA); Rabbit polyclonal antibody to the tri-peptide SKL were produced as previously described [60].

Donkey anti-mouse IgG conjugated to Alexa Fluor 680, goat anti-rabbit IgG conjugated to Alexa Fluor 680, donkey anti-mouse IgG conjugated to Alexa Fluor 488, donkey anti-rabbit IgG conjugated to Alexa Fluor 488, and donkey anti-mouse IgG conjugated to Alexa Fluor 546 were purchased from Invitrogen (Carlsbad, CA).

### Isolation and culture of monocyte-derived macrophages (MDMs)

The buffy coats used for PBMC isolation were derived from healthy volunteer blood donors. Human monocytes were isolated using Histopaque (Sigma-Aldrich). Briefly, the blood was diluted 1:1 with phosphate-buffered saline (PBS), placed under a layer of Histopaque and centrifuged for 22 min at 1800 rpm in a clinical centrifuge. Cells from the interphase layer were harvested, washed twice with serum-free RPMI, and then resuspended in RPMI1640 with 15% FBS, 1% penicillin and streptomycin (Invitrogen, Carlsbad, CA). The cells (2–4 million per well) were then seeded in 6-well plates that were pre-coated with poly-L-ornithine (Sigma, St. Louis, MO). After 4 hours, the cells were washed three times with warm RPMI medium before adding 2mL Differentiation medium (25 ng/mL macrophage colony-stimulatory factor (M-CSF) (Sigma, St. Louis, MO) in RPMI containing 2mM L-glutamine, 1% penicillin and streptomycin and 15% FBS) to each well. Cells were incubated for 7 days in this media (with media changes every 3 day) to allow differentiation of MDMs.

### Cell culture, transfection and virus infection

A549 and HEK293T cells from the American Type Culture Collection (Manassas, VA) were cultured in DMEM (Invitrogen) containing 10% heat-inactivated FBS, 4.5 g/liter D-glucose, 2 mM glutamine, 110 mg/liter sodium pyruvate at 37°C in a 5% CO_2_ atmosphere. Hela CD4+ (clone 1022) cells (NIH AIDS Reagent Program, Germantown, MD) were cultured in RPMI 1640 supplemented with 10% FBS and 1.0 mg/ml G418 (Geneticin, Gibco).

A549 and HEK293T cells were transfected with the expression plasmids using Lipofectamine 2000 (Invitrogen) and PerFectin (Genlantis) respectively as described by the manufacturers. When using miRNA mimics or anti-miRs, cells were transfected with Lipofectamine RNAiMAX (Invitrogen). HIV-1 infection in Hela CD4+ cells (pYU2, MOI = 10) or primary monocyte-derived macrophages (pYU2, MOI = 2) was performed under biosafety CL-3 conditions.

### Reporter constructs for miRNA target validation

To test whether miRNA mimics could silence predicted target genes, the entire 3’-untranslated regions (UTRs) of selected target genes were subcloned into the luciferase expression vector pMIR-REPORT-Luc (Ambion). Plasmids were constructed using polymerase chain reaction (PCR) and standard subcloning techniques. Sequence-verified full-length cDNAs of each PEX gene were used as templates to amplify the 3’-UTRs by PCR with primers listed in Table 2. The resulting PCR products were digested with HindIII and then subcloned immediately downstream of the luciferase cassette contained in the reporter plasmid pMIR-REPORT-Luc. The orientation of each 3’-UTR insert was determined by endonuclease digestion and all constructs were then verified by DNA sequencing.

**Table 2:** Oligonucleotide primers.

Primer name	Sequence
PEX2/3’-UTR-Forward	5-CTAT**AAGCTT**AAACTAAAATTGCTTCCTTTGAGG-3
PEX2/3’-UTR-Reverse	5-GCGT**AAGCTT**GATTATGCACTGCTGTTACT-3
PEX7/3’-UTR-Forward	5-CTAT**AAGCTT**CTGGGACTACAGTTTTCACCA-3
PEX7/3’-UTR-Reverse	5-GCGT**AAGCTT**ATTTATCACAGCAGTGATTAT-3
PEX11B/3’-UTR-Forward	5-CTAT**AAGCTT**CCTTCCGGTACAGGATAAG-3
PEX11B/3’-UTR-Reverse	5-GCGT**AAGCTT**GTCGATGAGCAAACTGAACTT-3
PEX13/3’-UTR-Forward	5-CTAT**AAGCTT**TATCTTTCATGTTTGCCTGC-3
PEX13/3’-UTR-Reverse	5-GCGT**AAGCTT**CAGATCAGAAAATTTTATTATTGAG-3
miR-500a-5p-Forward	5-TAATCCTTGCTACCTGGGTGAGA-3
miR-34c-3p-Forward	5-AATCACTAACCACACGGCCAGG-3
miR-93-3p-Forward	5-ACTGCTGAGCTAGCACTTCCCG-3
miR-381-3p-Forward	5-TATACAAGGGCAAGCTCTCTGT-3
miR-483-5p-Forward	5-AAGACGGGAGGAAAGAAGGGAG-3
Viperin-Forward	5-TGGTGAGGTTCTGCAAAGTAG-3
Viperin-Reverse	5-GTCACAGGAGATAGCGAGAATG-3
IRF1-Forward	5-CATGGCTGGGACATCAACAA-3
IRF1-Reverse	5-GTTCATGGCACAGCGAAAGTT-3
IFI6-Forward	5-GGTCTGCGATCCTGAATGGG-3
IFI6-Reverse	5-TCACTATCGAGATACTTGTGGGT-3
IFIT2-Forward	5-AGAAGCAGGCAATCACAGAAAA-3
IFIT2-Reverse	5-CTGAAACCGACCATAGTGGAAAT-3
OAS1-Forward	5-TGTCCCTCTCTAAATGCTGCTC-3
OAS1-Reverse	5-GGAAGCAGGAGGTCTCACCAG-3

Restriction endonuclease sites are bolded and underlined

### Luciferase reporter / β-galactosidase assay for miRNA target validation

The pMIR-REPORT miRNA Expression Reporter System (Ambion) was used to validate miRNA targets and conduct quantitative evaluations of miRNA function. The assay employs an experimental firefly luciferase-based reporter vector and an associated β-gal reporter control plasmid (pMIR-REPORT β-gal). The pMIR-REPORT Luciferase plasmid contains a firefly luciferase reporter gene upstream of a multiple cloning site for insertion 3’UTRs that contain predicted miRNA-binding sites in its 3’-UTR. By cloning a cDNA fragment with a miRNA target sequence into the pMIR-REPORT plasmid, expression of the luciferase reporter can be negatively regulated by miRNAs. β-galactosidase expression from the pMIR-REPORT β-gal was used to normalize variability due to differences in cell viability and/or transfection efficiency.

After 48 hours, lysates prepared from HEK293T cells transfected with pMIR-REPORT-Luciferase containing 3’-UTRs from different PEX genes (PEX2, PEX7, PEX11B or PEX13), pMIR-REPORT β-gal together with miRNA mimics were subjected to luciferase and β-gal assays. Briefly, growth medium was removed and cells were rinsed once with PBS. A minimal volume of 1X Reporter Lysis Buffer (RLB) (Promega) was added to each well and then the plates were rocked for several times to ensure complete coverage of the cells with RLB. Cells scraped from the wells were transferred to microcentrifuge tubes and placed on ice for 10 minutes. The microcentrifuge tubes were vortexed for 10–15 seconds and then centrifuged at 12,000 x g for 2 minutes at 4°C. The supernatant/cell lysates were transferred to new tubes and used immediately for assays or stored at -70°C.

For luciferase assays, 20 μl of cell lysate and 100 μl of Luciferase Assay Reagent (Promega) were mixed in microcentrifuge tubes and luminescence was measured using a model Synergy 4 Luminometer (BioTek). For β-Galactosidase assays, 150 μl of cell lysate (2:1 dilution to 1X RLB) was mixed with 150 μl of Assay 2X Buffer (Promega) and then incubated at 37°C for 30 minutes or until a faint yellow color had developed. The reactions were terminated with 1M sodium carbonate (500 μl) after which the absorbances were read at 420 nm. The relative luciferase activity was expressed as a ratio of luciferase activity to β-gal activity.

### Immunoblotting

Transfected or HIV-infected cells grown in 6-well plates were washed twice with cold PBS on ice and then lysed with RIPA buffer (50 mM Tris-HCl pH 7.4, 150 mM NaCl, 1% Triton x-100, 1% Sodium deoxycholate, 0.1% SDS, 1 mM EDTA) containing a cocktail of protease inhibitors. Lysates were incubated on ice for 30 minutes and then centrifuged at 14,000 x g for 15 minutes at 4°C after which protein concentrations in the supernatants were quantified using a Pierce BCA protein assay kit (Thermo Scientific). Equivalent amounts of total protein (20 μg) were resolved by SDS-PAGE and then transferred to polyvinylidene difluoride membranes (EMD Millipore) membranes for immunoblotting.

Membranes were blocked with 3% skim milk powder in PBS containing 0.1% Tween 20 (PBS-T) and then incubated overnight at 4°C or 3 hours at room temperature with appropriate primary antibodies diluted in 3% milk-PBS-T. After washing three times with PBS-T for 10 minutes each, fluorescent secondary antibodies (donkey anti-mouse IgG conjugated to Alexa Fluor 680 or goat anti-rabbit IgG conjugated to Alexa Fluor 680) diluted in PBS-T were used to detect the primary antibodies. After 1-hour incubation with the secondary antibodies, membranes were washed three times with PBS-T for 10 minutes each. Detection and quantification of the protein signals in the immunoblots was performed using a Licor Odyssey Infrared Imaging System (Lincoln, NE) using the protocol posted at http://biosupport.licor.com. Relative levels of PMP70, PEX2, PEX7, PEX11B, PEX13, PEX19, and catalase (normalized to actin) were determined using Odyssey Infrared Imaging System 1.2 Version software.

### Confocal and super-resolution microscopy

Hela CD4+ and A549 cells grown on coverslips were processed respectively for confocal or super-resolution microscopy at 48h post-transfection or infection. Cells were washed in PBS containing 0.5 mM Ca^2+^ and 1.0 mM Mg^2+^ and then fixed with 3% paraformaldehyde (for confocal imaging) or 1.5% electron microscopy grade paraformaldehyde (for super-resolution imaging) for 30 min at room temperature. Samples were then quenched with 50mM NH_4_Cl in PBS for 5 minutes at room temperature, washed three times with PBS, and then permeabilized with 0.2% Triton-X-100 for 5 min. Incubations with primary antibodies diluted (1:500–1000) in blocking buffer (3% BSA in PBS) were performed at room temperature for 2 hours followed by three washes in PBS containing 0.1% BSA. Samples were then incubated with secondary antibodies in blocking buffer for 1 hour at room temperature followed by three washes in PBS containing 0.1% BSA. Secondary antibodies were donkey anti-mouse/rabbit IgG conjugated to Alexa Fluor 488 and donkey anti-mouse IgG conjugated to Alexa Fluor 546 (Invitrogen).

For confocal microscopy, coverslips were mounted onto microscope slides using ProLong Gold antifade reagent with DAPI (Invitrogen), and samples were examined using an Olympus 1x81 spinning disk confocal microscope equipped with a 60x/1.42 oil PlanApo N objective. Confocal images were acquired and processed using Volocity 6.2.1 software.

For super-resolution microscopy, coverslips were mounted on slides pre-cleaned with acetone and ethanol using SlowFade Gold reagent mounting media (Invitrogen). Images were acquired using a DeltaVision OMX V4 structured illumination microscope (Applied Precision, GE) equipped with a 60x 1.42 oil PSF (PlanApo N) objective and immersion oil N = 1.514~1.516. Images were analyzed using Volocity 6.2.1 software.

### Quantification of peroxisomes

Z-stack images acquired using a confocal microscope were exported from Volocity 6.2.1 as an OEM.tiff file. The exported images were then processed using Imaris 7.2.3 software (Bitplane). Peroxisomes within polygonal areas that excluded the nucleus were quantified (quality and voxel). Within the selected regions, the absolute intensity/region volume of the peroxisomes were determined and then entered into a Microsoft Excel spreadsheet. The data were then analyzed using student’s t-test.

Where indicated, 0.125 μm optical sections acquired using an Applied Precision OMX super resolution microscope (with a 60X/1.42 Oil lens and three CMOS cameras) were also analyzed. The raw data were processed using Deltavision OMX SI image reconstruction and registration software and the final images were imported into Volocity 6.2.1 software as.dv files for quantification. In each cell, peroxisomes were selected based on the absolute pixel intensity in the corresponding channel and their numbers and volumes were then determined. Only those SKL/PMP70-positive structures with volumes between 0.001 and 0.05 μm^3^ were included for measurement.

### Immunohistochemistry and histochemistry

Formalin-fixed paraffin-embedded human brain was processed and tissue sections (10 μm) were prepared and labeled as described us previously [13, 61, 62]. Briefly, samples were deparaffinized by incubation for 1 hour at 60^°^C followed by one 10 min and 2 five min incubations in xylene baths through decreasing concentrations of ethanol to distilled water. Antigen retrieval was performed by boiling in 10mM sodium citrate (pH 6.0) 1 hr. Slides were blocked with HHFH buffer (1 mM HEPES buffer, 2% (v/v) horse serum, 5% (v/v) FBS, 0.1% (w/v) sodium azide in Hank’s balanced salt solution (HBSS)) for 4 hrs at room temperature. Slides were stained with hematoxylin and eosin (H&E). In addition, serial brain sections were immuno-labelled with antibodies to host proteins. Immunocytochemistry was performed with rabbit anti-Iba-1 (Wako Pure Chemical Industries Ltd., Osaka Japan), anti-thiolase or anti-PEX13 with appropriate secondary antibodies. For immunofluorescence studies, slides were incubated with a cocktail of rabbit anti-GFAP (DAKO, Carpenteria CA) or anti-Iba-1 (1:400) and anti-PEX13, overnight at 4°C. The primary antibodies were removed by three 5 min PBS washes and slides were incubated for three min in 0.22 micron filtered 1% (w/v) Sudan black in 70% ethanol and washed an additional 3 times in PBS. A cocktail of 1:500 Alexa 488 goat anti rabbit IgG, Alexa 568 goat anti mouse IgG for two hrs, washed 3 times in PBS stained with DAPI for 10 min, washed 3 times in PBS and mounted with Prolong gold antifade reagent. Slides were imaged with Wave FX spinning disc confocal microscope (Zeiss).

### QPCR analysis of miRNA expression

Total RNA including small RNA from HIV-infected Hela CD4+ cells or primary MDMs was purified using the miRNeasy Mini Kit (Qiagen) according to the manufacturer’s instructions. Mature miRNAs, certain small nucleolar RNAs and small nuclear RNAs (snoRNAs and snRNAs) were selectively reverse-transcribed into cDNA using miScript HiSpec buffer according to the instructions of miScript II RT Kit (Qiagen). Mature miRNAs, which are polyadenylated, were reverse transcribed into cDNA using oligo-dT primers. The oligo-dT primers included a 3’ degenerate anchor and a universal tag sequence on the 5’ end, allowing amplification of mature miRNA during the real-time PCR step.

The resulting cDNAs served as the template for real-time PCR analysis using miRNA-specific primers (forward primers, from IDT) and the miScript SYBR green PCR kit (Qiagen), which contains the miScript universal primer (reverse primer) and QuantiTect SYBR green PCR master mix. The amplification cycles consisted of an initial activation step at 95°C for 15 min, followed by 40 cycles of 15s at 94°C, 30s at 55°C and 30 s at 70°C. Fluorescence data were collected during the 70°C extension step. The miRNA targets and primers that were used in this study are listed in Table 2. As an internal control, levels of a small nuclear RNA RNU6B (a miScript PCR control provided in the miScript PCR starter kit (Qiagen)) were determined. Relative miRNA expression was normalized to RNU6B levels using the comparative cT (ΔΔcT) method. All miRNA expression studies were conducted using a Mx3005P (Stratagen, LaJolla, CA) thermocycler.

### Accession numbers

Microarray data were deposited into the NCBI GEO database (Accession number GSE97611).

## Supporting information

S1 Fig Specificity of the HAND-associated miRNAs. HEK293T cells were co-transfected with luciferase reporter plasmids (pMIR-REPORT-Luciferase) containing 3’-UTRs from PEX2, PEX7, PEX11B and PEX13) in forward (5’-3’) or reverse orientations (3’-5’), a transfection control reporter plasmid (pMIR-REPORT-β-gal) and miRNA mimics for miR-500a-5p (A), miR-34c-3p (B), miR-93-3p (C) or miR-381-3p (D). After 48 hours, cell lysates were subjected to luciferase and β-gal assays. N = 3. Error bars represent standard error of the mean. From the data it can be see that each miRNA only suppresses one reporter construct. Specifically: miR-500a-5p suppresses expression of PEX2 (A); miR-34c-3p suppresses expression of PEX7 (B); miR-93-3p suppresses expression of PEX11B (C); and miR-381-3p suppresses expression of PEX13 (D), Key to plasmids: pMIR-Vec = pMIR-REPORT-Luciferase; pMIR-KLF4 = pMIR-REPORT-Luciferase with 3’ UTR of KLF4 downstream from luciferase cassette; pMIR-PEX2 = pMIR-REPORT-Luciferase with 3’ UTR of PEX2 downstream from luciferase cassette; pMIR-PEX7 = pMIR-REPORT-Luciferase with 3’ UTR of PEX7 downstream from luciferase cassette; pMIR-PEX11B = pMIR-REPORT-Luciferase with 3’ UTR of PEX11B downstream from luciferase cassette; pMIR-PEX13 = pMIR-REPORT-Luciferase with 3’ UTR of PEX13 downstream from luciferase cassette.
(TIF)

S2 Fig Knockdown of one PEX protein can affect the stabilities of other PEX proteins. **A.** Individual siRNAs against PEX7, PEX11B, PEX13 or PEX19 were transfected into HEK293T cells for 48 hours and then levels of peroxisomal proteins were determined by immunoblotting with corresponding antibodies. B. The average relative levels of peroxisomal proteins (compared to actin) from 3 independent experiments are shown. Error bars represent standard error of the mean.
(TIF)

S3 Fig HIV-1 infection causes loss of peroxisomal proteins in Hela CD4+ cells. Hela CD4+ cells (clone 1022) were infected with HIV-1 (pYU2, MOI = 10.0) for 72 hours and then subjected to immunoblot analyses with antibodies to PMP70, PEX2, PEX7, PEX11B, PEX13, HIV-1 p24 and actin. The relative levels of peroxisomal proteins (compared to actin) from 3 independent experiments were averaged and plotted. Error bars represent standard error of the mean.
(TIF)
