# Supplementary figures and images for: MicroRNAs upregulated during HIV infection target peroxisome biogenesis factors: Implications for virus biology, disease mechanisms and neuropathology
Source: PLoS Pathog. 2017 Jun 8;13(6):e1006360. doi: 10.1371/journal.ppat.1006360 (PMC5464672; doi:10.1371/journal.ppat.1006360)

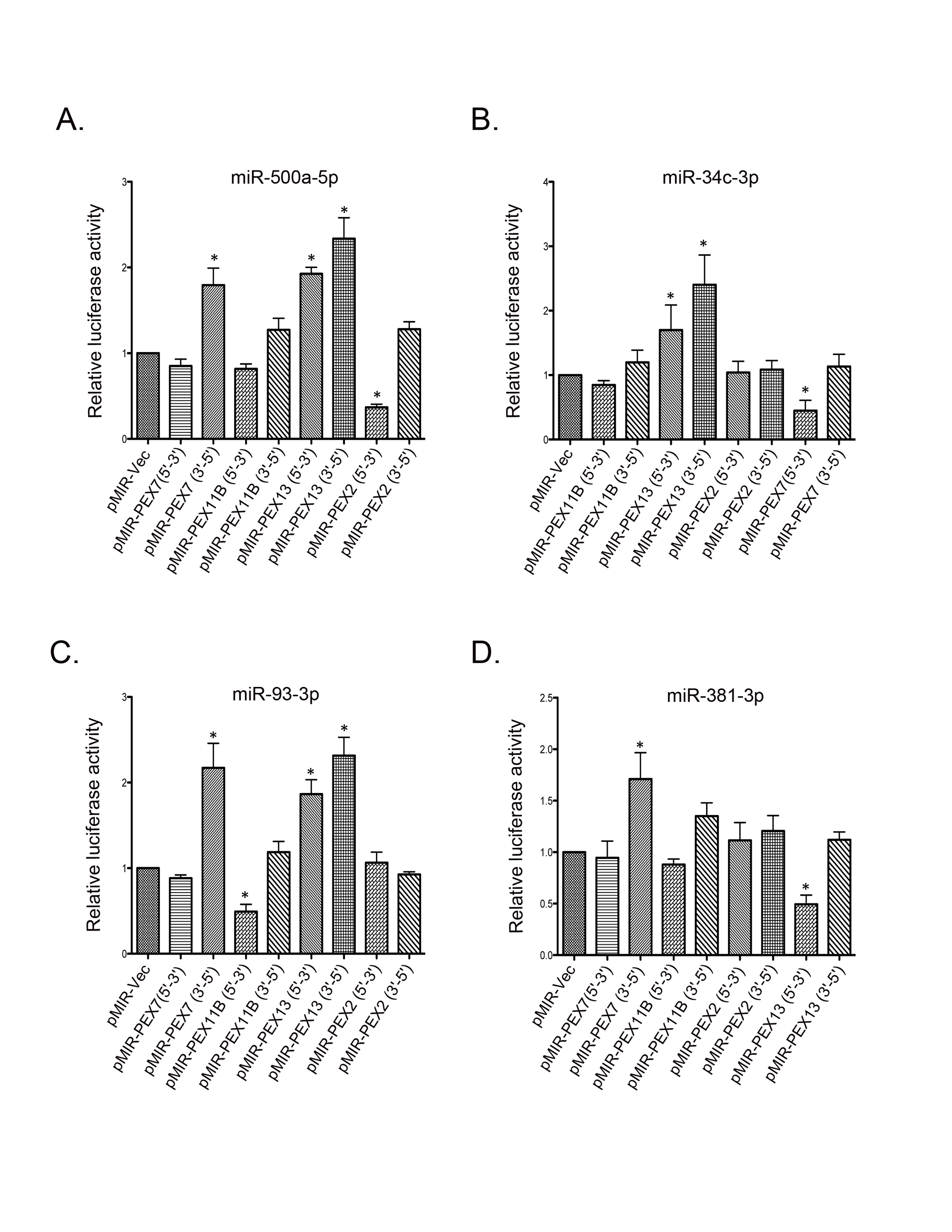

Supplement: S1 Fig — HEK293T cells were co-transfected with luciferase reporter plasmids (pMIR-REPORT-Luciferase) containing 3’-UTRs from PEX2, PEX7, PEX11B and PEX13) in forward (5’-3’) or reverse orientations (3’-5’), a transfection control reporter plasmid (pMIR-REPORT-β-gal) and miRNA mimics for miR-500a-5p (A), miR-34c-3p (B), miR-93-3p (C) or miR-381-3p (D). After 48 hours, cell lysates were subjected to luciferase and β-gal assays. N = 3. Error bars represent standard error of the mean. From the data it can be see that each miRNA only suppresses one reporter construct. Specifically: miR-500a-5p suppresses expression of PEX2 (A); miR-34c-3p suppresses expression of PEX7 (B); miR-93-3p suppresses expression of PEX11B (C); and miR-381-3p suppresses expression of PEX13 (D), Key to plasmids: pMIR-Vec = pMIR-REPORT-Luciferase; pMIR-KLF4 = pMIR-REPORT-Luciferase with 3’ UTR of KLF4 downstream from luciferase cassette; pMIR-PEX2 = pMIR-REPORT-Luciferase with 3’ UTR of PEX2 downstream from luciferase cassette; pMIR-PEX7 = pMIR-REPORT-Luciferase with 3’ UTR of PEX7 downstream from luciferase cassette; pMIR-PEX11B = pMIR-REPORT-Luciferase with 3’ UTR of PEX11B downstream from luciferase cassette; pMIR-PEX13 = pMIR-REPORT-Luciferase with 3’ UTR of PEX13 downstream from luciferase cassette. (TIF) [file ppat.1006360.s001.tif]

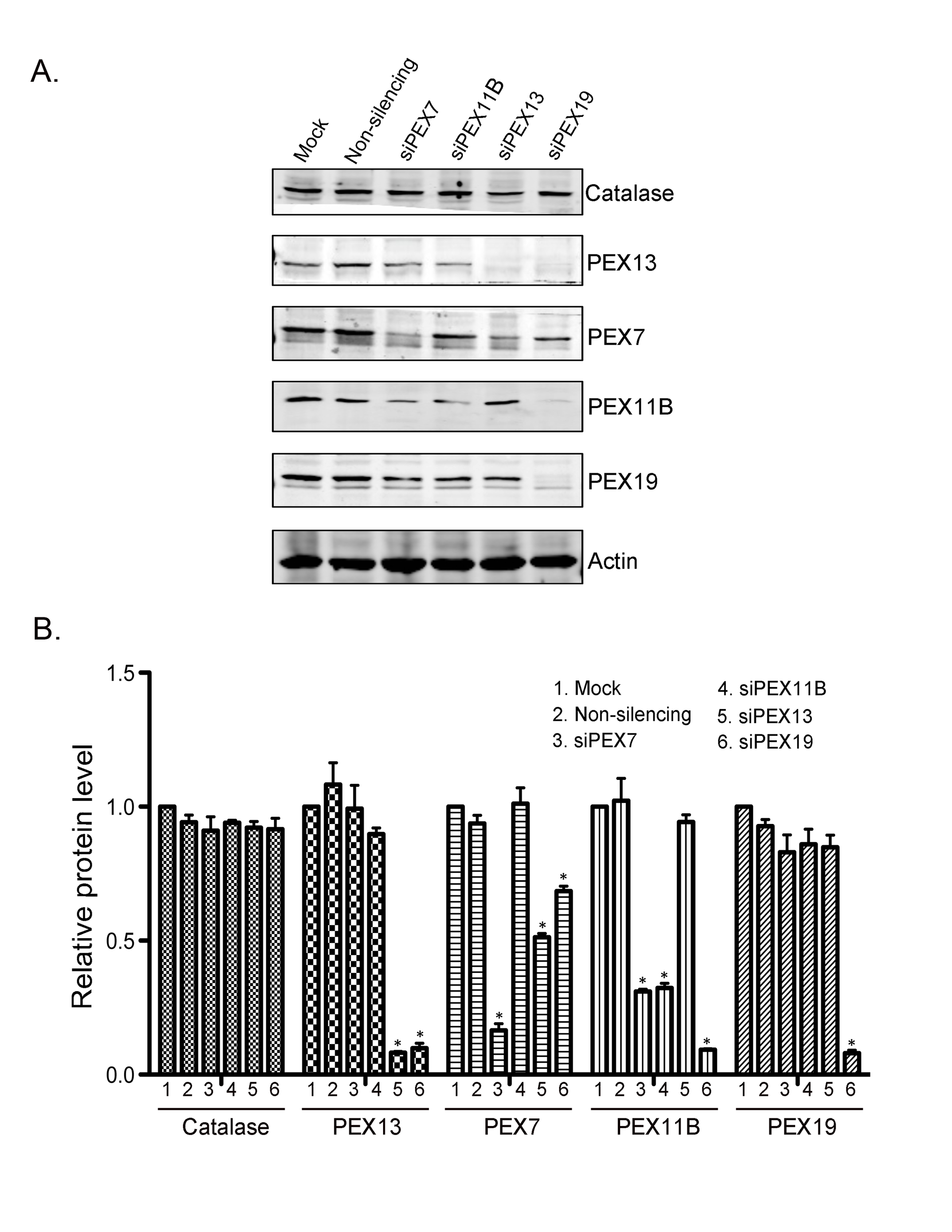

Supplement: S2 Fig — A. Individual siRNAs against PEX7, PEX11B, PEX13 or PEX19 were transfected into HEK293T cells for 48 hours and then levels of peroxisomal proteins were determined by immunoblotting with corresponding antibodies. B. The average relative levels of peroxisomal proteins (compared to actin) from 3 independent experiments are shown. Error bars represent standard error of the mean. (TIF) [file ppat.1006360.s002.tif]

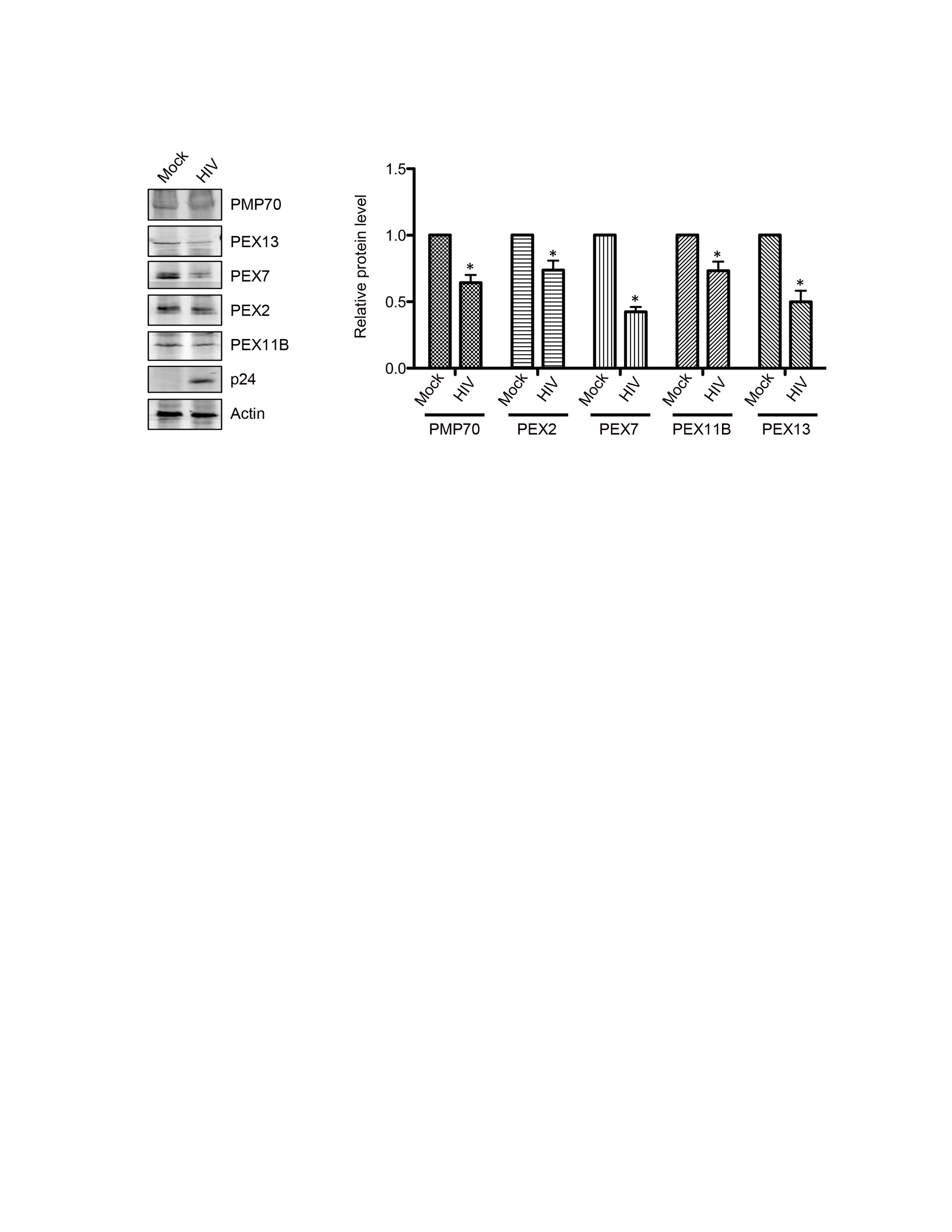

Supplement: S3 Fig — Hela CD4+ cells (clone 1022) were infected with HIV-1 (pYU2, MOI = 10.0) for 72 hours and then subjected to immunoblot analyses with antibodies to PMP70, PEX2, PEX7, PEX11B, PEX13, HIV-1 p24 and actin. The relative levels of peroxisomal proteins (compared to actin) from 3 independent experiments were averaged and plotted. Error bars represent standard error of the mean. (TIF) [file ppat.1006360.s003.tif]
